# Prevalence of Transfusion-Transmitted Infections (HCV, HIV, Syphilis and Malaria) in Blood Donors: A Large-Scale Cross-Sectional Study

**DOI:** 10.3390/pathogens11070726

**Published:** 2022-06-26

**Authors:** Talal Alharazi, Tawfique K. Alzubiery, Jerold C. Alcantara, Husam Qanash, Abdulrahman S. Bazaid, Malik A. Altayar, Abdu Aldarhami

**Affiliations:** 1 Department of Medical Laboratory Science, College of Applied Medical Sciences, University of Ha’il, Hail 55476, Saudi Arabia; j.alcantara@uoh.edu.sa (J.C.A.); h.qanash@uoh.edu.sa (H.Q.); ar.bazaid@uoh.edu.sa (A.S.B.); 2 Department of Medical Microbiology and Immunology, Faculty of Medicine and Health Sciences, Taiz University, Taiz P.O. Box 6803, Yemen; 3 Molecular Diagnostics and Personalized Therapeutics Unit, University of Ha’il, Hail 55476, Saudi Arabia; 4 Department of Medical Laboratory, Faculty of Medical and Health Sciences, Taiz University Al-Turbah Branch, Al-Turbah P.O. Box 6803, Yemen; tawk2020@gmail.com; 5 Department of Medical Laboratory Technology, Faculty of Applied Medical Sciences, University of Tabuk, Tabuk 71491, Saudi Arabia; maltayar@ut.edu.sa; 6 Department of Medical Microbiology, Qunfudah Faculty of Medicine, Umm Al-Qura University, Al-Qunfudah 21961, Saudi Arabia; ahdarhami@uqu.edu.sa

**Keywords:** blood donors, HCV, HIV, syphilis, malaria

## Abstract

Blood plays a major role in transmitting infectious diseases such as hepatitis C virus (HCV), human immunodeficiency virus (HIV), hepatitis B virus (HBV), syphilis, malaria, and many others. Thus, this study sought to evaluate the distribution of HCV, HIV, syphilis, and malaria among blood donors in Yemen. This is a cross-sectional study, conducted on blood donors at the national center in Yemen. Blood donors’ specimens were serologically tested for the presence of anti-HCV and anti-HIV antibodies, as well as anti-*Treponema pallidum*, anti-*Plasmodium falciparum*, and anti-*Plasmodium vivax*. A total of 16,367 donors were included in this study. Based on the donor’s occupation, the study showed that the relative seroprevalence of anti-HCV Ab among the donors was statistically significant, and relatively high prevalence was found among military donors (2.8%). Positive HIV antibody tests were only reported in 33 male donors (0.2%), who were mostly manual workers. A remarkably high prevalence of anti-*Treponema pallidum* was observed among manual workers (3.1%). There was a statistically significant difference in the distribution of anti-malaria Ab based on residency and age groups. This study revealed that the prevalence of HCV, HIV, syphilis, and malaria among donors was 2.0%, 0.2%, 2.4%, and 0.7%, respectively. Further genotyping studies are necessary to provide a complete picture of the prevalence of transfusion-transmitted infections (TTIs).

## 1. Introduction

Blood transfusion is a mandatory and therapeutic procedure that plays a crucial role in saving patient lives. Globally, it is estimated that 92 million people donate blood annually [1]. Roughly, 1.6 million of these blood units are discarded because of the presence of infectious agents [2]. Blood transfusion carries the risk of transmitting major infections, such as hepatitis B virus (HBV), hepatitis C virus (HCV), human immunodeficiency virus (HIV), syphilis, cytomegalovirus (CMV), herpes simplex virus (HSV), and Epstein–Barr virus (EBV), along with toxoplasmosis and malaria [3]. Therefore, collected blood units must be serologically tested to prevent the transmission of infections caused by such pathogens, as per the recommendation of the World Health Organization (WHO) [4].

Infections due to HBV, HCV, and HIV are major public health concerns [4]. According to WHO, the prevalence of HCV worldwide is 854.09 per 100,000 people. Within the WHO divisions, Africans (2503.61 per 100,000) and Europeans (450.21 per 100,000) have the highest and lowest ratio, respectively. At the country level, the Netherlands have a rate of 25,370 per 100,000 people, whereas Cambodia has a rate of 14,670 per 100,000 people. [5]. In 2019, newly infected individuals with chronic hepatitis C and hepatitis B reached over 1.5 million. Also, 290,000 [230,000–580,000] people died from infections related to hepatitis C in 2019 [6]. Additionally, recent data have indicated that hepatitis B and C kill about 1.1 million people and infect another 3.0 million annually [6].

Chronic hepatitis, cirrhosis, and hepatocellular carcinoma have been associated with blood-borne hepatitis C viruses [7]. This virus is widespread worldwide, ranging from 0.2% to 40% in different regions [8]. HCV infections are believed to affect nearly 170 million people worldwide [9]. Approximately 3.5 million new cases occur annually [9], and about 21.3 million people are estimated to be carrying the HCV virus in the Eastern Mediterranean region [10]. Around two-thirds of viral hepatitis mortality and morbidity are caused by HCV, which is more prevalent in Egypt, where the prevalence is 13 to 22% [11]. Various global epidemiological studies have revealed that HCV prevalence varies greatly around the world. The hepatitis C virus has been found to be endemic in several countries including Egypt, and several studies have confirmed its endemic status [12].

HCV has infected 2.2% of the global population [13]. The prevalence of HCV in Arabian Gulf countries is as follow: 0.30% in Bahrain, 0.41% in Oman, 1.06% in Qatar, 1.45% in Kuwait, 1.63% in Saudi Arabia, and 1.64% in the United Arab Emirates [14]. Expatriate populations, including Egyptians, exhibited a higher prevalence of HCV, as high as 78.6% in people who have received multiple transfusions and as high as 74.6% in people who use drugs [14]. In north Arabian African countries, the prevalence of HCV ranged from 0.7% in Tunisia to 7.7% in Morocco [11].

As previously reported, the prevalence of HCV among healthy volunteers and blood donors in Yemen was 1.7% and 2.7%, respectively [15]. Recently, the prevalence of hepatitis C infections among blood donors has been 1.0% to 1.6% [16,17]. On the other hand, it was estimated that the prevalence of HCV-Ab among the intermediate-risk populations in Yemen ranges from 0.5 to 3.5% compared to 21.3% among populations with liver-related conditions [11].

HIV is a global health problem with a considerable socioeconomic impact [18]. There were almost 1.7 million cases of HIV in 2019, the lowest number since 1990 and down by 23% from the peak in 2010. However, this is still far from the global target of fewer than 500,000 new infections by the year 2020 [6]. In Yemen, it was reported that the prevalence of HIV ranges from 0.30% to 0.60% [13,19], and syphilis from 0.60% to 0.75% with the number of cases expected to increase [20]. In addition, 17.1 million people aged 15–49 are expected to be infected with *T. pallidum* annually [6,21]. Transfusion-transmitted infections (TTIs) in Yemen are one of the major public health problems. Moreover, there is limited current information on blood transfusion infections and the impact of this problem on the Yemeni population. We still lack comprehensive studies dealing with this issue due to the absence of a nationwide registry, hospital studies, or reports, all of which would yield valuable information regarding its trend. Therefore, several comprehensive studies are still necessary. Thus, the modest goal of this study is to assess the prevalence of HCV, HIV, syphilis, and malaria among blood donors from different Yemeni communities.

## 2. Results

A total of 16,367 blood donors were involved in the study, with a mean age of 30.19 years (17–63 and SD: 7.5). The vast majority of them were aged between 26–35 years (48.7%), followed by 16–25 years (30.8%). Most of the donors were male (99.1%), and 153 (0.9%) were female. Participants were primarily manual workers (5440; 33.2%) and professional workers (5193; 31.7%) whereas the minority of the donors were military personnel (2422; 14.8%). Concerning the place of residence, a large number of blood donors in the study were from the capital city (8820; 53.9%), followed by Azal (6438; 39.3%). Moreover, the majority of the blood donors were replacements at 11,804 (72.1%), compared to 4563 (27.9%) who were volunteers (Table 1 and Table 2).

Overall, the prevalence of anti-HCV, anti-HIV, anti-*Treponema pallidum* (syphilis), and anti-malarial antibodies among blood donors with seroprevalence was 2%, 0.2%, 2.4%, and 0.7%, respectively (Table 1 and Table 2). The prevalence of HCV, HIV, syphilis, and malaria among replacement donors were 1.8%, 0.2%, 2.6%, and 0.6%, respectively, compared to 2.4%, 0.2%, 1.8%, and 0.7% among volunteers who were seropositive for HCV, HIV, syphilis, and malaria. The difference in the distribution of HCV (X^2^ = 6.8 and *p* = 0.009) and syphilis (X^2^ = 9.5 and *p* = 0.002) among the replacement and volunteer blood donors was statistically significant (Table 1 and Table 2). The outcome of the logistic regression analysis revealed a statistically significance association between occupation and HCV, HIV, and syphilis. Significant differences also exist between the type of donor and HCV, between the donor’s residency and syphilis, and between the age groups with malaria (Table 3).

### 2.1. HCV

The difference in the seroprevalence of anti-HCV among donors in relation to their occupations was found to be statistically significant (*X^2^* = 13.6 and *p* = 0.003) (Table 1). A high proportion was found among military donors (2.8%), followed by manual workers (1.9%), and students (1.9%), whereas the lowest proportion was observed among professional workers (1.6%). Similarly, the difference in the results of HCV among volunteers (2.4%) and replacement donors (1.8%) was found to be statistically significant (*X^2^* = 6.8 and *p* = 0.009) (Table 1). A high prevalence (3.5%) of HCV was found among donors residing in the Tehama region, but the lowest prevalence was found in the Azal region (1.8%). Moreover, no statistical significance was observed in the prevalence of HCV among donors according to their residency (*X^2^* = 2.8 and *p* = 0.585) or age group (*X^2^* = 2.5 and *p* = 0.654) (Table 1).

### 2.2. HIV

The seroprevalence of anti-HIV among blood donors was 0.2% (33) (Table 1). All female donors tested negative for anti-HIV. The differences in the HIV results among blood donors according to their occupation were found to be statistically significant (*X^2^* = 9.0 and *p* = 0.029). The greatest proportion in terms of anti-HIV antibodies was found among manual workers, with 0.3% and the least was among students (0.1%) and professional workers (0.1%) (Table 1). Moreover, 0.1% of blood donors residing in the Alganad and Azal regions were seropositive for HIV, compared to 0.2% and 0.9% of residents in the capital city of Sana’a and the Tehama region, respectively (Table 1). This HIV prevalence was found to be statistically insignificant. Similarly, no statistically significant difference was found in the HIV prevalence of the different age groups. All blood donors older than 45 years were seronegative for HIV, compared to 0.1%, 0.2%, and 0.3% of blood donors aged 16–25, 36–45, and 26–35 years old who were seropositive (Table 1). In addition, an analogous HIV prevalence (0.2%) was found for both volunteers and replacement donors (Table 1).

### 2.3. Syphilis

The study revealed that 2.4% of males and 1.3% of females were seropositive for anti-*Treponema pallidum* (Table 2). Notably, a statistically significant difference in anti-*Treponema pallidum* prevalence among donors was observed in relation to their occupation (*X^2^* = 30.9 and *p* = 0.001), residency (*X^2^* = 35.0 and *p* = 0.001), age groups (*X^2^* = 12.6 and *p* = 0.013), and type of donor (*X^2^* = 9.5 and *p* = 0.002) (Table 2). In terms of occupation, manual workers (3.1%) had the highest prevalence of anti-*Treponema pallidum* antibodies, whereas the least was observed among students (1.5%). The prevalence was notably found elevated among donors residing in Tehama (10.5%) and among the population aged more than 55 years old (4.7%) (Table 2).

### 2.4. Malaria

The overall prevalence of antibodies to malaria parasites was 0.7% (Table 2). The distribution of anti-malaria Ab was found to be statistically significant in terms of residency (*X^2^* = 21.0 and *p* = 0.001) and age group (*X^2^* = 26.4 and *p* = 0.001), with a prevalence ranging from 0.4% (among students and donors residing in Sana’a) to 4.7% (among donors aged more than 55 years old). The difference in prevalence was observed to be statistically insignificant in terms of donor type (*X^2^* = 0.8 and *p* = 0.367), and donor occupation (*X^2^* = 5.8 and *p* = 0.123), with a prevalence ranging from 0.4% to 0.8% (Table 2).

### 2.5. Logistic Regression Analysis (LRA) of Sociodemographic Characters Related to HCV, HIV, Syphilis, and Malaria

All factors that were significant at *p* < 0.05 in the univariate analysis were subjected to a binary logistic regression model by using the standard method. The final “best-fit” model contained one factor that was independently associated with positive HCV status: donors who were volunteers (AOR = 0.73; *p* = 0.009). Being military personnel (AOR = 3.21; *p* = 0.014) was a high-risk factor for HIV infection. In addition, factors that were associated with syphilis included donors who were students (AOR = 1.983; *p* = 0.001), and military (AOR = 1.74; *p* = 0.001), donors who lived in regions other than Tehama, and the replacement donors (AOR = 1.414; *p* = 0.006). Furthermore, all donor age groups older than 25 years were associated with high malaria risk factors (Table 3).

## 3. Discussion

The present study highlights the prevalence of TTIs among blood donors in Yemen. A large proportion of donors (99.1%) were male. Various studies also reported a larger number of male donors than female donors [22,23]. Regarding the gender profile of blood donors, WHO has revealed that 33% of blood donations have been given by women. Of 113 reporting countries, 15 countries reported that less than 10% of blood donations were from female donors [4]. A recent study has reported that social pressure and personal obligations were the foremost reasons for blood donation in men, whereas altruism or community responsibility were identified to be the main motives among women [24]. The same study has also showed that the higher deferral rates among women were due to medical unsuitability, anemia, pregnancy, breast-feeding, low body weight, low deposits of iron, low blood pressure, and other health problems [24]. Also, the limited number of female donors may be related to culture and belief, including the fact that because women regularly lose blood during menstruation, they should refrain from donating blood [25].

The majority of the blood donors in this study were replacements (72.1%), compared to 27.9% who were volunteers. This is inconsistent with the Ethiopian report [26] that 30.8% of donors were replacements, and 69.2% were volunteers. These variations could be attributed to several reasons, including the center locations and public awareness of the importance of donation. The present study illustrated that 2.4% of volunteers and 1.8% of replacements had HCV (Table 1), which is higher than that found in India [27], where 0.1% of both volunteers and replacements had HCV. Saba et al. [27] also reported a lower prevalence of syphilis among replacements (0.10%) and volunteers (0.71%) than in the current study (2.6% and 1.8%, respectively). Further analysis revealed that the prevalence of HCV among volunteers was significantly higher, but also significantly lower in terms of syphilis prevalence. Various international studies have observed that the replacement system of donation was found to have higher incidence of unsuitable donors as compared to voluntary donors. Individuals who give blood under pressure from the patient’s family are less likely to reveal any reasons why they may be unsuitable as donors. For this reason, they pose a higher risk to the safety of the blood supply [28].

### 3.1. HCV

The findings of the present study are consistent with previous reports [29,30] conducted among the general population in Yemen. However, lower proportions were observed in other counties, such as the United Arab Emirates (0.24%), Kuwait (0.44%), Qatar (0.51%), and Saudi Arabia (1.65%) [14], Algeria (0.3%), Mauritania, (1.1%), Morocco, (0.8%), and Tunisia (0.6%), [31]. A higher prevalence was reported in Egypt (9.02%) [32], Nigeria (15.1%) [33], and Sudan (3.4%) [34].

The current study revealed a higher prevalence (2.0%) of anti-HCV antibodies compared to studies conducted in the same area (Sana’a) (1.0% and 1.6%) [16,19]. However, this study has a lower prevalence than the studies of Alodini (3.0%) [35] and Saghir et al. (2.4%) [20]. Moreover, a recent study by Osuji et al., [33] reported a higher prevalence of HCV among donors according to their occupation and age groups, unlike the present study.

A lower prevalence of HCV in terms of age groups has been reported [36,37]. A higher prevalence (11.35%) among older adults (more than 45 years old) was found in the study of Abebe et al. [38] as compared to the present study (≤1.6%). Moreover, some interesting observations were noted in the present study. Despite the minority of military personnel as blood donors (14.8%), this group had the highest prevalence of anti-HCV Ab with 2.8%. Similarly, donors from the Tehama region were more likely to be infected with HCV at 3.5% which was also associated with an age of more than 55 years old.

### 3.2. HIV

The present study revealed a lower prevalence (0.2%) of anti-HIV Ab than previous studies conducted in Sana’a city, with a prevalence of 0.3 to 0.6% [19,20]. A recent study illustrated an approximate similarity in the prevalence of anti-HIV [39]. Similarly, Makroo et al. [40] reported parallel results (0.24%) in north India, but both are less than those reported by Bazie et al. (0.7%) [34]. In addition, the results of the current study showed a higher prevalence (0.2%) than the findings of AlMutairi et al. (0.04%) [41], and Abdelaziz (0.05%) [42]. Likewise, the prevalence in this study was lower than in other studies conducted in Sudan (0.7%) [34] and Mauritania (1.2%) [43].

Similar to the observation in terms of anti-HCV prevalence, it was noted that donors who are resident of Tehama were found to have increased reactivity to anti-HIV ab (0.9%) as compared to other regions. Further investigation is needed to fully understand the situation.

### 3.3. Syphilis

As compared to the present study, Saghir et al., [20] documented a lower prevalence of syphilis in Sana’a which reported a prevalence of 0.75%. Comparable findings were also reported in Ethiopia (0.73%) [36] and Bengal, 0.72% [44]. In contrast to this study, a lower prevalence of anti-*Treponema pallidum* was also reported in Saudi Arabia (0.2%) [45], Nepal (0.02%) [46], and Jordan (0.02%) [47]. The prevalence of syphilis in this study ranged from 1.8% to 4.7% among various age groups. This was found to be quite similar to that previously reported [48]. A lower prevalence (0.0% to 0.5%) was reported by Abate and Wolde [36].

Surprisingly, the largest proportion (4.7%) of reactive syphilis was found among donors who were older than 55 years despite the low number of participants from this age group. The lowest percentage (1.8%) was donors aged between 16–25 years. Consistent with the results for HCV and HIV, donors residing in the Tehama region showed the highest prevalence of reactive syphilis (10.5%) even with their low number of participants (114). Participants who worked as military personnel had the highest level (3.0%) of antibodies against syphilis.

### 3.4. Malaria

The prevalence of anti-malarial antibodies among blood donors in the current study is 0.7%, which was consistent with the findings of Hoque et al. (0.76%) [49]. Meanwhile, a lower prevalence was reported by Ehsan et al. in Pakistan (0.11) [50] and Patel et al. [46] in Nepal (0.02%). Several studies conducted in Ethiopia (4.1%) [51] and Tanzania (8%) [52] reported high levels of anti-malarial antibodies. Ezeonu et al. [53] in Abuja, Nigeria, recorded a higher prevalence (45.8%), whereas a lower prevalence (0.11%) was observed in Pakistan [54].

This study displayed that 0.7% of all males and females had malaria. This agrees with the findings by Bartonjo et al. [48] in Kenya, in which 0.7% of male and 0.6% of female donors had malaria parasites. This is in comparison to the results of a recent study performed in Tanzania by Morona et al. [52] which showed a high prevalence of malaria for all females (5.88%) and males (8.27%), whereas Baffour et al. [55] in Ghana reported that 9.0% of females and 39.0% of males were seropositive for malaria. Interestingly, the prevalence increased with age (0.6% among the 16–25 age group and 4.7% among the age group older than 55 years old). This was inconsistent with the findings of Bartonjo et al. [48], who reported that no malaria parasites were noticed among age groups older than 25 years old. In addition, Bartonjo et al. [48] identified no malarial parasites among blood donors in all occupation categories except for students. The present study, however, reported a prevalence of 0.4% among students and 0.8% among professionals.

### 3.5. Logistic Regression Analysis (LRA) of the Sociodemographic Characters Related to HCV, HIV, Syphilis, and Malaria

Professional workers were determined to be not statistically associated with positive HCV. In contrast, Alzubiry et al. stated that professional workers, such as nurses, medical doctors, and laboratory technicians, were more susceptible to HBV and HCV infections due to their frequent contacts with infected patients [56]. Moreover, military and volunteer donors were high-risk factors for HIV, HCV, and syphilis infections, respectively. This is in agreement with a previous study that revealed that HBV, HCV, and HIV are more prevalent among military and volunteer donors [17]. Studies worldwide indicate that donors’ populations that are composed mostly of students produce a safer blood supply [48]. This study revealed that donors who were students were more likely to have positive antibodies to syphilis. The possible reason for this association is not clear, and further evaluation is needed. Notably, the age groups older than 25 years were associated with high malaria risk factors. This may be because individuals more than 25 years old are more susceptible to the infection than the younger age group [57], probably due to their greater involvement in nocturnal activities, which may be social or occupational in nature that enhance their contact with mosquitoes.

### 3.6. Limitations of the Study

Although this study investigated TTIs in a large number of donors, it has some limitations. Given the extremely low proportion of female donors who participated in the study (below 1%), the discussion of gender differences was limited. This study presents vital data for public health and provides relevant information in relation to TTIs among blood donors in Yemen. Factors including differences in health care systems, the economic status of the country in which the study was carried out, and the scale of the risk factors for contracting TTIs could have caused these differences. Blood safety and availability remain a challenge in transfusion centers, and TTIs continues to be a primary concern. Hence, information generated from such study is pivotal in monitoring and evaluating the trends and scale of TTIs among donor populations, as they allow effective intervention strategies to be developed and implemented.

## 4. Materials and Methods

### 4.1. Data Collection and Processing

This study employed a descriptive cross-sectional design and, was conducted on 16,367 blood donors who attended at the National Blood Transfusion and Research Centre in Sana’a, Yemen, from January 2019 to December 2020. A self-administered structured questionnaire, capturing respondents’ age, gender, residence, occupation, and previous blood donation history was filled in prior to donation. A trained public health specialist was present to conduct face-to-face interviews with blood donors who had difficulty with reading and writing.

Blood donors were first classified into two main groups: volunteers and replacements, the latter referring to those who specifically donate blood for their family members or friends. Then they were classified into four groups according to their occupation: students, provisional workers, manual workers, and military personnel. Professional workers are individuals with educational qualifications, such as teachers, engineers, health workers, employees, etc. Manual workers are people who have low educational qualifications or are freelancers, such as carpenters, plumbers, drivers, waiters, etc. Blood pressure, hemoglobin levels and body temperature were measured, and a general health checkup was performed on all donors. Healthy people aged over 16 years old with a body weight of more than 45 kg were eligible to donate blood and were included in this study.

### 4.2. Electrochemiluminescence (ECL)

Trained laboratory technicians collected five milliliters of blood from each blood donor, following a standard aseptic technique. All serum specimens were centrifuged and tested for anti-HCV and HIV Ag-Ab employing the ECL technique and using the Cobas e-411 analyzer (Roche ELECSYS^®^ 2010 GmbH; Germany). The specificity of the technique employed was 99.85% with a sensitivity of 100% [58,59].

### 4.3. Enzyme Immunoassay (EIA)

Serum specimens were analyzed via the EIA technique for anti-HIV antibodies by using Gen screenTM Ultra HIV Ag-Ab kit (BioRad Diagnostics, France). For anti-HCV Ab, the MonolisaTM Anti-HCV plus, Version 3 (BioRad Diagnostics, France) was used. The Bio-Rad Microplate Reader Machine read the results of the reaction of all the tested specimens. The sensitivity and specificity of the tests were 94% and 100%, respectively [60]. Rapid immunochromatographic kits were used, such as the standard diagnostics kit (MT Promedt Consulting GmbH, Germany) to screen for the presence of anti-*Plasmodium falciparum,* anti-*Plasmodium vivax* malarial parasites, and syphilis. The sensitivity and specificity of the test kits were 92.9% and 98.4%, respectively [61].

### 4.4. Statistical Analysis

The proportions of seropositive individuals among the total donor population were calculated for males only as they represented 99% of the sample. These were expressed as percentages to determine the prevalence. Appropriate descriptive statistics were used to describe the socio-demographic variables and other characteristics of the donor population. All statistical analysis were performed with the SPSS 20 software package. The *p*-values were calculated by employing the chi-squared test software package or the Fisher exact test, and the prevalence of infectious diseases in the population was statistically compared. Statistical significance was set at a *p*-value < 0.05. Furthermore, multivariable logistic regression methods were employed to identify potential risk factors linked to infectious disease.

## 5. Conclusions

The prevalence of HCV, HIV, syphilis, and malaria among blood donors in Yemen was 2.0%, 0.2%, 2.4%, and 0.7%, respectively. Further studies are indispensable to provide a complete picture of the disease prevalence, including the genotyping of viral hepatitis. It is recommended that the public be offered more health education about the risk factors of HCV, HIV, syphilis, and malaria parasite infections. The data generated could increase public awareness and assist in understanding the occurrence and risk factors related to TTIs allowing public health policies and interventions to be improved.

## Figures and Tables

**Table 1:** Seropositivity of anti-HCV and anti-HIV Abs in relation to the demographic characteristics of donor population.

Demographic Characteristics		Total *n* = 16,367		HCV				HIV			
				Reactive 320 (2.0)		Non-Reactive 16,047 (99.2%)		Reactive 33 (0.2)		Non-Reactive 16,334 (99.8)	
		*n*	%	*n*	%	*n*	%	*n*	%	*n*	%
**Occupation**	Students	3312	20.2	63	1.9	3249	98.1	4	0.1	3308	99.9
	Professional worker	5193	31.7	83	1.6	5110	98.4	6	0.1	5187	99.9
	Military	2422	14.8	69	2.8	2353	97.2	4	0.2	2418	99.8
	Manual workers	5440	33.2	105	1.9	5335	98.1	19	0.3	5421	99.7
	*X^2^*			13.6				9.0			
	*p*-value			0.003 *				0.039 **			
	Capital city	8820	53.9	181	2.1	8639	97.9	22	0.2	8798	99.8
	Alganad	845	5.2	17	2.0	828	98.0	1	0.1	844	99.9
	Azal	6438	39.3	115	1.8	6323	98.2	9	0.1	6429	99.9
**Residency**	Shepah	150	0.9	3	2.0	147	98.0	0	0.0	150	100.0
	Tehama	114	0.7	4	3.5	110	96.5	1	0.9	113	99.1
	*X^2^*			2.8				5.4			
	*p*-value			0.585				0.248			
	16–25	5045	30.8	106	2.1	4939	97.9	7	0.1	5038	99.9
	26–35	7968	48.7	159	2.0	7809	98.0	20	0.3	7948	99.7
	36–45	2689	16.4	45	1.7	2644	98.3	6	0.2	2683	99.8
**Age groups**	45–55	601	3.7	9	1.5	592	98.5	0	0.0	601	100.0
	More than 55	64	0.4	1	1.6	63	98.4	0	0.0	64	100.0
	*X^2^*			2.5				3.4			
	*p*-value			0.654				0.499 **			
	Volunteer	4563	27.9	110	2.4	4453	97.6	11	0.2	4552	99.8
**Type of donors**	Replacement	11,804	72.1	210	1.8	11,594	98.2	22	0.2	11,782	99.8
	*X^2^*			6.8				0.5			
	*p*-value			0.009 *				0.484			

* Statistically significant; *X^2^*, chi-square; *p* value < 0.05, significant; *n*, number; %, percentage; ** Fisher exact test.

**Table 2:** Seropositivity of anti-malarial and anti-*Treponema pallidum* in relation to the demographic characteristics of donor population.

Demographic Characteristics		Total *n* = 16,367		Syphilis				Malaria			
				Reactive 387 (2.4%)		Non-Reactive 15,980 (97.6%)		Reactive 107 (0.7%)		Non-Reactive 16,260 (99.3%)	
		*n*	%	*n*	%	*n*	%	*n*	%	*n*	%
**Occupation**	Students	3312	20.2	51	1.5	3261	98.5	13	0.4	3299	99.6
	Professional worker	5193	31.7	97	1.9	5096	98.1	42	0.8	5151	99.2
	Military	2422	14.8	72	3.0	2350	97.0	14	0.6	2408	99.4
	Manual workers	5440	33.2	167	3.1	5273	96.9	38	0.7	5402	99.3
	*X^2^*			30.9				5.8			
	*p*-value			0.001 *				0.123			
	Capital city	8820	53.9	215	2.4	8605	97.6	36	0.4	8784	99.6
	Alganad	845	5.2	16	1.9	829	98.1	4	0.5	841	99.5
	Azal	6438	39.3	140	2.2	6298	97.8	64	1.0	6374	99.0
**Residency**	Shepah	150	0.9	4	2.7	146	97.3	2	1.3	148	98.7
	Tehama	114	0.7	12	10.5	102	89.5	1	0.9	113	99.1
	*X^2^*			35.0				21.0			
	*p*-value			0.029 *				0.0.031 **			
	16–25	5045	30.8	93	1.8	4952	98.2	29	0.6	5016	99.4
	26–35	7968	48.7	195	2.4	7773	97.6	41	0.5	7927	99.5
	36–45	2689	16.4	81	3.0	2608	97.0	27	1.0	2662	99.0
**Age groups**	45–55	601	3.7	15	2.5	586	97.5	7	1.2	594	98.8
	More than 55	64	0.4	3	4.7	61	95.3	3	4.7	61	95.3
	*X^2^*			12.6				26.4			
	*p*-value			0.027 **				0.37 **			
	Volunteer	4563	27.9	81	1.8	4482	98.2	34	0.7	4529	99.3
**Type of donors**	Replacement	11,804	72.1	306	2.6	11,498	97.4	73	0.6	11,731	99.4
	*X^2^*			9.5				0.8			
	*p*-value			0.002 *				0.367			

* Statistically significant; *X^2^*, chi-square; *p* value < 0.05, significant; *n*, number; %, percentage; ** Fisher exact test.

**Table 3:** Final model of factors associated with positive HCV, HIV, syphilis and malaria among blood donors.

Characteristics	Adjusted odds Ratio (AOR)	95% Confidence Interval	*p-*Value
**HCV reactive**			
Professional worker	0.88	0.764, 1.438	0.615
Volunteer	0.73	0.580–0.926	0.009
Manual worker	Ref		
**HIV reactive**			
Military	3.21	1.260, 8.151	0.014
Manual worker	Ref		
**Syphilis reactive**			
students	1.983	1.442, 2.726	0.001
Military	1.74	1.345, 2.255	0.001
Manual worker	Ref		
Capital City	4.708	2.542, 8.720	0.001
Alganad	5.36	2.864, 10.017	0.001
Azal	5.93	2.717, 12.933	0.001
Shepam	4.74	1.480, 12.933	0.009
Tehama	Ref		
Replacement	1.414	1.103, 1.813	0.006
Volunteer	Ref		
**Malaria positive**			
16–25 years old	Ref		
26–35 years old	4.17	1.052, 16.554	0.034
36–45 years old	4.85	1.432, 16.416	0.010
46—55 years old	8.507	2.523, 28.675	0.004
>55	9.51	2.867, 31.539	0.001

## Data Availability

Data that support the findings of this study are available within the article, and from the corresponding author upon request.

## References

[1] Mohammed Y., Bekele A. (2016). Seroprevalence of transfusion transmitted infection among blood donors at Jijiga blood bank, Eastern Ethiopia: Retrospective 4 years study. BMC Res. Notes.

[2] World Health Organization (2012). Blood Donor Selection: Guidelines on Assessing Donor Suitability for Blood Donation.

[3] Noubiap J.J., Joko W.Y., Nansseu J.R., Tene U.G., Siaka C. (2013). Sero-epidemiology of human immunodeficiency virus, hepatitis B and C viruses, and syphilis infections among first-time blood donors in Edéa, Cameroon. Int. J. Infect. Dis..

[4] World Health Organization (2018). Blood Safety and Availability.

[5] Kasraian L., Hosseini S., Marzijarani M.S., Ebrahimi A., Ashkani-Esfahani S. (2020). The Prevalence of Hepatitis C Infection in Blood Donors: A Meta-Analysis and Systematic Review. Iran. Red Crescent Med. J..

[6] World Health Organization (2021). Global Progress Report on HIV, Viral Hepatitis and Sexually Transmitted Infections.

[7] Dwyre D.M., Fernando L.P., Holland P.V. (2011). Hepatitis B, hepatitis C and HIV transfusion-transmitted infections in the 21st century. Vox Sang..

[8] Alavian S.M. (2009). Hepatitis C Infection in Iran: A Review Article. Iran. J. Clin. Infect. Dis..

[9] Gao X., Cui Q., Shi X., Su J., Peng Z., Chen X., Lei N., Ding K., Wang L., Yu R. (2011). Prevalence and trend of hepatitis C virus infection among blood donors in Chinese mainland: A systematic review and meta-analysis. BMC Infect. Dis..

[10] Fallahian F., Najafi A. (2011). Epidemiology of hepatitis C in the Middle East. Saudi J. Kidney Dis. Transpl..

[11] World Health Organization—Regional Office for the Eastern Mediterranean (2020). Epidemiology of Hepatitis C Virus in the WHO Eastern Mediterranean Region: Implications for Strategic Action.

[12] Sievert W., Altraif I., Razavi H.A., Abdo A., Ahmed E.A., Alomair A., Amarapurkar D., Chen C.H., Dou X., El Khayat H. (2011). A systematic review of hepatitis C virus epidemiology in Asia, Australia and Egypt. Liver Int..

[13] Global Burden of Hepatitis C Working Group (2004). Global burden of disease (GBD) for hepatitis C. J. Clin. Pharmacol..

[14] Mohamoud Y.A., Riome S., Abu-Raddad L.J. (2016). Epidemiology of hepatitis C virus in the Arabian Gulf countries: Systematic review and meta-analysis of prevalence. Int. J. Infect. Dis..

[15] Sallam T.A., Tong C.Y.W., Cuevas L.E., Raja’A Y.A., Othman A.M., Al-Kharsa K.R. (2003). Prevalence of blood-borne viral hepatitis in different communities in Yemen. Epidemiol. Infect..

[16] Al-Zubiery T.K.A., Alharazi T., Alsumairy H., Jabir A.A., Muckbil M.J., Saleh M.A., Al-Shibani E.A.A. (2017). Sero-prevalence of Anti- HCV among Yemenis Blood Donors Attending National Blood Transfusion and Research Centre in Sana’a: Yemen. Int. Blood Res. Rev..

[17] Tawfique A.-Z., Talal A., Hafez A., Adel A.-Z. (2017). Sero-Prevalence of HBs Ag, HCV and HIV among Blood Donors in Three Blood Bank Centers in Sana’a city: Yemen. J. Biotechnol. Biomed. Sci..

[18] United Nations Office on Drugs and Crime (2019). HIV Prevention, Treatment, Care and Support for People Who Use Stimulant Drugs.

[19] Al-Zubiery T.K., Alsumairy H., Alharazi T., Al-Zubeiry A., Jabir A.A., Saleh M.A., Muckbil M.J. (2018). Seroprevalence of Human Immunodeficiency Virus (HIV) among Blood Donors Attending National Blood Transfusion and Research Center in Sana’a City, Yemen: Warning Sign. Int. STD Res. Rev..

[20] Saghir S.A.M., Alsalahi O.S.A., Zabad A.A.M., Al-Hassan F.M. (2012). HIV and Syphilis among Blood Donors in Sana’a, Yemen. Biohealth Sci. Bull..

[21] Tucker J.D., Cohen M.S. (2011). China’s syphilis epidemic: Epidemiology, proximate determinants of spread, and control responses. Curr. Opin. Infect. Dis..

[22] Valerian D.M., Mauka W.I., Kajeguka D.C., Mgabo M., Juma A., Baliyima L., Sigalla G.N. (2018). Prevalence and causes of blood donor deferrals among clients presenting for blood donation in northern Tanzania. PLoS ONE.

[23] Siraj N., Achila O.O., Issac J., Menghisteab E., Hailemariam M., Hagos S., Gebremeskel Y., Tesfamichael D. (2018). Seroprevalence of transfusion-transmissible infections among blood donors at National Blood Transfusion Service, Eritrea: A seven-year retrospective study. BMC Infect. Dis..

[24] Kasraian L., Ashkani-Esfahani S., Foruozandeh H. (2021). Reasons of under-representation of Iranian women in blood donation. Hematol. Transfus. Cell Ther..

[25] Mremi A., Yahaya J.J., Nyindo M., Mollel E. (2021). Transfusion-Transmitted Infections and associated risk factors at the Northern Zone Blood Transfusion Center in Tanzania: A study of blood donors between 2017 and 2019. PLoS ONE.

[26] Heyredin I., Mengistie B., Weldegebreal F. (2019). Sero-prevalence of transfusion-transmittable infections and associated factors among blood donors in Eastern Ethiopia: An Institutional-based cross-sectional study. SAGE Open Med..

[27] Bommanahalli B., Javali R., Mallikarjuna S., Gouda K., Siddartha K., Shashikala K.P. (2014). Seroprevalence of hepatitis B and hepatitis C viral infections among blood donors of Central Karnataka, India. Int. J. Med. Sci. Public Health.

[28] WHO (2009). Safe Blood and Blood Products: Safe Blood Donation Module 1.

[29] Chaabna K., Kouyoumjian S.P., Abu-Raddad L.J. (2016). Hepatitis C Virus Epidemiology in Djibouti, Somalia, Sudan, and Yemen: Systematic Review and Meta-Analysis. PLoS ONE.

[30] Al-Hatheq A., Abakar A., Al-Ofairi B.A. (2019). Sero-prevalence of hepatitis Cvirus infections among blood donors and clinical visitors in Amran governorate, Yemen. Eur. J. Pharm. Med. Res..

[31] Fadlalla F.A., Mohamoud Y.A., Mumtaz G.R., Abu-Raddad L.J. (2015). The epidemiology of hepatitis C virus in the Maghreb region: Systematic review and meta-analyses. PLoS ONE.

[32] Hakami N.Y. (2021). The Most Common Causes of Transfusion-Transmitted Diseases among Blood Donors in the Middle Eastern States. J. Pharm. Res. Int..

[33] Osuji A.I., Duru G.C., Kinjir H. (2021). Detection of Hepatitis C virus antibodies among healthy blood donors at a tertiary hospital in Nigeria. Microbes Infect. Dis..

[34] Bazie E., Ali M., Hamza H., Magzoub O., Siddig M., Haroun E., Fathalla M., Khalid M.F. (2015). Sero-Prevalence of HIV and Syphilis Infections among Blood Donors at Kosti Teaching Hospital-White Nile State-Sudan. Int. J. Curr. Microbiol. Appl. Sci..

[35] Alodini A.Q. (2012). Prevalence of Hepatitis B Virus (HBV) and Hepatitis C Virus (HCV) Infections among Blood Donors at Al-Thawra Hospital Sana’a City-Yemen. Yemeni J. Med. Sci..

[36] Abate M., Wolde T. (2016). Seroprevalence of Human Immunodeficiency Virus, Hepatitis B Virus, Hepatitis C Virus, and Syphilis among Blood Donors at Jigjiga Blood Bank, Eastern Ethiopia. Ethiop. J. Health Sci..

[37] Alqahtani S.M., Alsagaby S.A., Mir S.A., Alaidarous M., Bin Dukhyil A., Alshehri B., Banawas S., Alturaiki W., Alharbi N.K., Azad T.A. (2021). Seroprevalence of Viral Hepatitis B and C among Blood Donors in the Northern Region of Riyadh Province, Saudi Arabia. Healthcare.

[38] Abebe M., Alemnew B., Biset S. (2020). Prevalence of Hepatitis B Virus and Hepatitis C Virus Among Blood Donors in Nekemte Blood Bank, Western Oromia, Ethiopia: Retrospective 5 Years Study. J. Blood Med..

[39] Naskar S., Nandy S., Basu K., Basu R. (2014). Study of Seroprevalence of HIV, Hepatits B and C And Syphilis Among Blood Donors In A Tertiary Care Hospital, Kolkata. IOSR J. Dent. Med. Sci..

[40] Makroo R.N., Hegde V., Chowdhry M., Bhatia A., Rosamma N.L. (2015). Seroprevalence of infectious markers & their trends in blood donors in a hospital based blood bank in north India. Indian J. Med. Res..

[41] AlMutairi H.H., AlAhmari M.M., Al-Zahran B.H., Abbas I.S., Al Ghamdi J.A., Raja A.Y., Sallam T.A. (2016). Prevalence of serological markers and nucleic acid for blood-borne viral infections in blood donors in Al-Baha, Saudi Arabia. J. Infect. Dev. Ctries..

[42] Abdelaziz M.O. (2020). Prevalence of Transfusion Transmissible Infection among Healthy Blood Donors at Dongola Specialized Hospital, Sudan, 2010–2015. Sudan J. Med. Sci..

[43] Boushab B.M., Limame O.C.M.M., Zahra F.M.F., Mamoudou S., Darnycka B.M.R., Saliou S.M. (2017). Estimation of seroprevalence of HIV, hepatitis B and C virus and syphilis among blood donors in the hospital of Aïoun, Mauritania. Pan Afr. Med. J..

[44] Mondal R., Koley S., Aggarwal I., Kumar N. (2019). Seroprevalence of Transfusion Transmitted Infections among the Blood Donors and the Trends of TTI in Last Three Years in a Tertiary Care Teaching Hospital in Durgapur. J. Evid. Based Med. Healthc..

[45] Alcantara J., Alenezi F., Ali O. (2018). Seroprevalence and trends of markers of transfusion transmissible infections among blood donors: A 3-year hospital based-study. Int. J. Community Med. Public Health.

[46] Patel A.K., Jana D., Jana N., Chaurasiya A., Prasad S., Shah N., Bhaskar R., Gupta R. (2018). Prevalence of HBV Infection among the Blood Donors of Southern Nepal: A Three Year Retrospective Study. Int. J. Health Sci. Res..

[47] Hroob A.M.A., Saghir S.A.M., Almaiman A.A., Alsalahi O.S.A., Al-Wajeeh A.S., Al-Shargi O.Y.A., Al-Balagi N., Mahmoud A.M. (2020). Prevalence and Association of Transfusion Transmitted Infections with ABO and Rh Blood Groups among Blood Donors at the National Blood Bank, Amman, Jordan. Medicina.

[48] Bartonjo G., Oundo J., Ng’ang’a Z. (2019). Prevalence and associated risk factors of transfusion transmissible infections among blood donors at Regional Blood Transfusion Center Nakuru and Tenwek Mission Hospital, Kenya. Pan Afr. Med. J..

[49] Hoque M., Islam M., Begum H., Rahman M., Rahman S., Al Mamun M., Chowdhury F., Hossain M. (2010). Prevalence of Malaria Parasites Among Blood Donors in Selected Hospitals of Dhaka City. J. Dhaka Med. Coll..

[50] Ehsan H., Wahab A., Shafqat M.A., Sana M.K., Khalid F., Abdullah S.M., Jaan A., Sheikh M.M., Muneeb A., Ehsan S. (2020). A Systematic Review of Transfusion-Transmissible Infections Among Blood Donors and Associated Safety Challenges in Pakistan. J. Blood Med..

[51] Alemu G., Mama M. (2016). Assessing ABO/Rh Blood Group Frequency and Association with Asymptomatic Malaria among Blood Donors Attending Arba Minch Blood Bank, South Ethiopia. Malar. Res. Treat..

[52] Morona D., Msemwa B., Zinga M.M., Mushi M.F., Mirambo M.M., Mshana S.E. (2017). Asymptomatic malaria and associated factors among blood donors in Mwanza, Tanzania. Tanzan. J. Health Res..

[53] Ezeonu C.M., Adabara N., Garba S.A., Kuta F.A., Ewa E.E., Oloruntoba P.O., Atureta Z. (2019). The risk of transfusion transmitted malaria and the need for malaria screening of blood donors in Abuja, Nigeria. Afr. J. Clin. Exp. Microbiol..

[54] Iram S., Saeed M., Hussain S., Mobeen A., Jabbar R., Aslam M. (2016). Burden of Malarial Parasitemia among Healthy Population of Lahore. Prof. Med. J..

[55] Antwi-Baffour S., Kyeremeh R., Amoako A.P., Annison L., Tetteh J.O., Seidu M.A. (2019). The Incidence of Malaria Parasites in Screened Donor Blood for Transfusion. Malar. Res. Treat..

[56] Alzubiery T., Alharazi T., Sumairi H., Jabir A., Muckbil M., Saleh M. (2017). Prevalence of HBsAg and Anti-HBc among Blood Donors at the National Blood Transfusion and Research Center, Sana’a, Yemen. World J. Pharm. Med. Res..

[57] World Health Organization, Malaria Control Unit, United Nations Children’s Fund (UNICEF) (2003). The Africa Malaria Report 2003.

[58] Mühlbacher A., Sauleda S., Piron M., Rietz R., Permpikul P., Klinkicht M., Gloeck M., Imdahl R. (2019). A multicentre evaluation of the Elecsys^®^ HIV Duo assay. J. Clin. Virol..

[59] Esteban J.I., van Helden J., Alborino F., Bürgisser P., Cellerai C., Pantaleo G., Eiras A., Rodriguez M.I., Ghisetti V., Gleich M. (2013). Multicenter evaluation of the Elecsys^®^ anti-HCV II assay for the diagnosis of hepatitis C virus infection. J. Med. Virol..

[60] Tang W., Chen W., Amini A., Boeras D., Falconer J., Kelly H., Peeling R., Varsaneux O., Tucker J.D., Easterbrook P. (2017). Diagnostic accuracy of tests to detect Hepatitis C antibody: A meta-analysis and review of the literature. BMC Infect. Dis..

[61] Meena M., Joshi D., Joshi R., Sridhar S., Waghdhare S., Gangane N., Kalantri S.P. (2009). Accuracy of a multispecies rapid diagnostic test kit for detection of malarial parasite at the point of care in a low endemicity region. Trans. R. Soc. Trop. Med. Hyg..

